# Prevalence of leptospirosis among soldiers: A systematic review

**DOI:** 10.1371/journal.pntd.0012927

**Published:** 2025-03-17

**Authors:** Pavlo Petakh, Valentyn Oksenych, Oleksandr Kamyshnyi

**Affiliations:** 1 Department of Biochemistry and Pharmacology, Uzhhorod National University, Uzhhorod, Ukraine; 2 Department of Clinical Science, Broegelmann Research Laboratory, University of Bergen, Bergen, Norway; 3 Department of Microbiology, Virology, and Immunology, I. Horbachevsky Ternopil National Medical University, Ternopil, Ukraine; Gulu University, UGANDA

## Abstract

Leptospirosis is a bacterial disease that spreads through water and soil contaminated with infected animal urine. Soldiers have a higher risk of infection because they often work in wet and muddy conditions. This systematic review examines how common leptospirosis is among military personnel. Studies published between January 2000 and November 2024 were collected from PubMed, Web of Science, and Scopus, following PRISMA guidelines. The review included studies that used laboratory tests to confirm leptospirosis cases in soldiers. Out of 67 studies, three met the inclusion criteria. These studies were conducted in Malaysia, Hawaii, and Honduras, with sample sizes between 488 and 1,000 soldiers. The infection rate ranged from 1.4% to 16.2%, with higher rates in tropical regions. Leptospirosis is often underdiagnosed in military personnel because symptoms are similar to other diseases, and testing is not always available. More awareness, better protective measures, and improved laboratory tests are needed to prevent infections. This review highlights the importance of better surveillance and health strategies for soldiers at risk of leptospirosis.

## Author summary

Leptospirosis is a bacterial disease that spreads through contaminated water and soil, often affecting people who work in wet and muddy conditions. Soldiers are at high risk of infection, especially in tropical regions, where the disease is more common. In this study, we reviewed research on leptospirosis in military personnel to understand how often it occurs and why it is a concern. We analyzed studies published between 2000 and 2024, focusing on those that investigated the seroprevalence among soldiers. Out of 67 studies, only three met our criteria. These studies were conducted in Malaysia, Hawaii, and Honduras, with seroprevalence ranging from 1.4% to 16.2%. We found that leptospirosis is often overlooked in military settings because its symptoms resemble other illnesses, and proper testing is not always available. Our findings highlight the need for better awareness, improved protective measures, and more accessible diagnostic tools to prevent infections among soldiers. By improving surveillance and health strategies, we can help protect military personnel from this potentially serious disease.

## 1. Introduction

Leptospirosis, first described as Weil’s disease by German physician Adolf Weil in 1886, is an infectious disease characterized by splenomegaly, jaundice, and nephritis [1]. Transmission occurs through direct contact with the urine of infected animals or exposure to contaminated water and soil [2]. Military personnel are particularly at risk due to their activities, which often involve prolonged exposure to water and mud—conditions conducive to the transmission of *Leptospira* [3].

The military significance of leptospirosis was first recognized during World War I, when epidemic outbreaks of Weil’s disease, marked by jaundice, were reported among soldiers engaged in trench warfare [4]. Troops from Britain, Germany, France, and Belgium experienced significant morbidity, with similar accounts from Italian and Canadian forces. The causative agent, Leptospira, was identified during this period through parallel research by military medical services in Germany, France, Britain, and Japan, which had previously described the disease [5].

During World War II, leptospirosis persisted as a challenge for military operations, despite the shift away from trench warfare [6]. Epidemics were documented among British soldiers at the Normandy beachhead and German troops in the Charente Valley. Few medical testing centers and little knowledge about leptospirosis symptoms made it difficult to report cases at that time. Later wars, such as the Korean War and military missions in Malaya and Indochina, showed that the disease remained an important problem for military health [7].

Leptospirosis remains a concern in modern conflicts, such as the ongoing Russo-Ukrainian War. A study by Ogorodniychuk et al. in 2022 reported that 6 contract service members and 40 mobilized personnel contracted leptospirosis, representing 32% of all cases [8–10]. Additionally, some cases have been reported among civilians, with suspected transmission occurring in bomb shelters [11]. Given the long history and ongoing importance of leptospirosis in military groups, this review aims to gather and summarize current data on the prevalence of leptospirosis in soldiers.

## 2. Methods

This systematic review was conducted following the Preferred Reporting Items for Systematic Reviews and Meta-Analyses (PRISMA) guidelines [12], with adherence to the standards outlined in S1 File PRISMA Checklist. The review aimed to examine the prevalence of leptospirosis among soldiers by systematically identifying and synthesizing relevant studies. This review was prospectively registered with PROSPERO, the international prospective register of systematic reviews, under the registration number CRD42024574351.

### 2.1. Outcomes and inclusion/exclusion criteria

The inclusion criteria focused on studies that investigated soldiers, military personnel, or defense forces and reported on leptospirosis prevalence using serological diagnostic tests (MAT, ELISA, or similar). Eligible studies included cross-sectional, cohort, or case-control designs that provided sufficient data to calculate prevalence rates and confidence intervals. Studies published between January 2000 and November 2024 were considered, with no restrictions on geographic location. Articles published in English or in other languages that could be translated were included.

Studies were excluded if they focused on non-military populations or mixed populations without separate analysis for soldiers. We also excluded studies on highly specific military subgroups (e.g., marines, personnel working exclusively with animals) that may not represent the general military population. Reviews, editorials, commentaries, and case series without population-level prevalence data were also excluded. Additionally, studies without clear diagnostic criteria for leptospirosis or those without available full text or necessary data for analysis were omitted. Duplicate publications, overlapping datasets, and animal or laboratory studies unrelated to humans were not included.

### 2.2. Study selection

Relevant studies were identified from PubMed, Web of Science, and Scopus using subject heading terms and free-text words. A comprehensive search strategy is presented in S1 File Search Strategy. All identified studies were evaluated for validity and reliability. While systematic and non-systematic literature reviews were excluded, their reference lists were screened for additional studies. To ensure consistency during the selection process, a pilot round of title and abstract screening was conducted using a random sample of 30 titles. Two independent reviewers (P.P. and O.K.) evaluated these titles to determine their eligibility and refine the inclusion criteria. A high measure of inter-rater agreement (>90%) was achieved, following which the remaining titles were divided and screened independently by the two reviewers. Full-text screening was subsequently conducted independently by two reviewers (P.P. and V.O.). Any disagreements were resolved through discussion with a third reviewer (O.K.). Studies that met the inclusion criteria after the full-text screening were included in the review.

### 2.3. Data extraction, synthesis, and presentation

Data extraction was conducted independently by two reviewers (P.P. and O.K.) using a predesigned data extraction sheet in a standardized Excel format. Extracted data included study characteristics (e.g., first author, year of publication), geographical context (country or area), sample size, diagnostic methods, and prevalence estimates. Any discrepancies in the extracted data were resolved through discussion and consensus.

### 2.4. Assessment of study quality

The quality of the included studies was assessed using the Newcastle-Ottawa Scale (NOS), adapted for cross-sectional studies [13]. Each study was evaluated across three domains: selection, comparability, and outcome. The selection domain assessed the representativeness of the sample, sample size justification, and ascertainment of exposure. The comparability domain assessed how well confounding factors, like geographic region or military subgroup, were controlled. The outcome domain focused on the accuracy of diagnostic methods and the sufficiency of prevalence reporting. Each domain was scored, with a maximum of 10 points per study. Studies scoring seven or more points were considered high quality, those scoring between four- and six-points medium quality, and those scoring less than four points low quality.

## 3. Result

**Fig 1:**
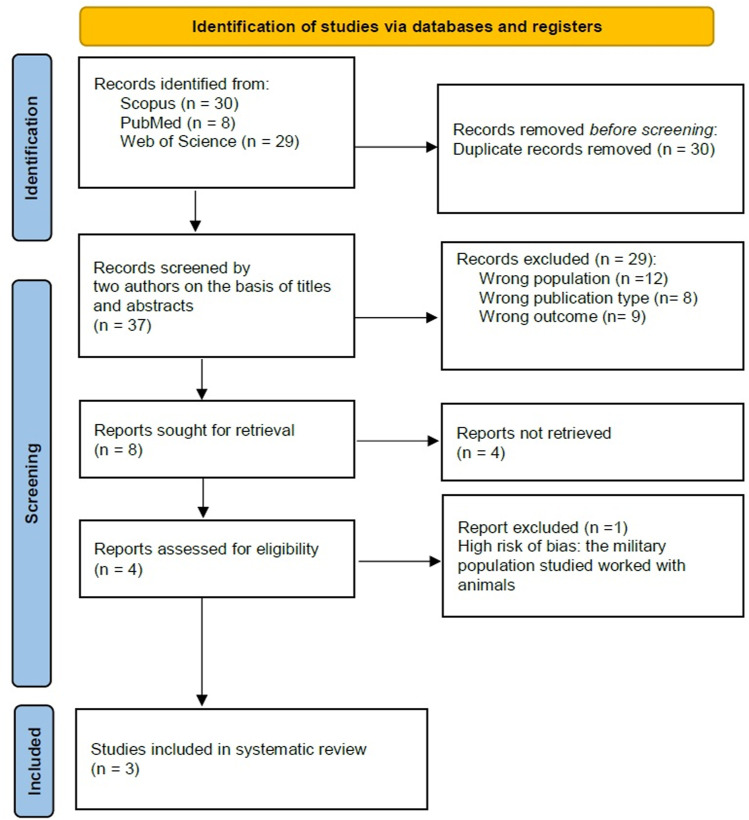
PRISMA flow diagram of the study selection process.

A total of 67 studies were identified through the initial database search (PubMed: 8, Web of Science: 29, Scopus: 30). After removing duplicates (n = 30), 37 unique records were screened based on titles and abstracts. Following this, 4 full-text articles were assessed for eligibility, of which three studies met the inclusion criteria and were included in this systematic review. The PRISMA flow diagram outlining the study selection process is presented in Fig 1.

### 3.1. Characteristics of included studies

The included studies were conducted across various geographic regions, including Northeastern Malaysia, Hawaii, and Honduras. Sample sizes ranged from 488 to 1,000 soldiers, with diagnostic methods including the microscopic agglutination test (MAT) and enzyme-linked immunosorbent assay (ELISA). Prevalence rates varied significantly across studies, from 1.4% to 16.2%. Detailed characteristics of the included studies are summarized in Table 1.

**Table 1:** Prevalence of leptospirosis among military personnel based on diagnostic methods and geographic location.

Author(s) and Year	Location	Sample Size	Diagnostic Method	Prevalence (%)
Sara et al. (2020) [34]	Northeastern Malaysia	616	ELISA + MAT	16.2
Lettieri et al. (2004) [35]	Hawaii	488	MAT	1.4
Chao et al. (2022) [36]	Honduras	1,000	ELISA	11.6

### 3.2. Risk of bias and quality assessment

**Fig 2:**
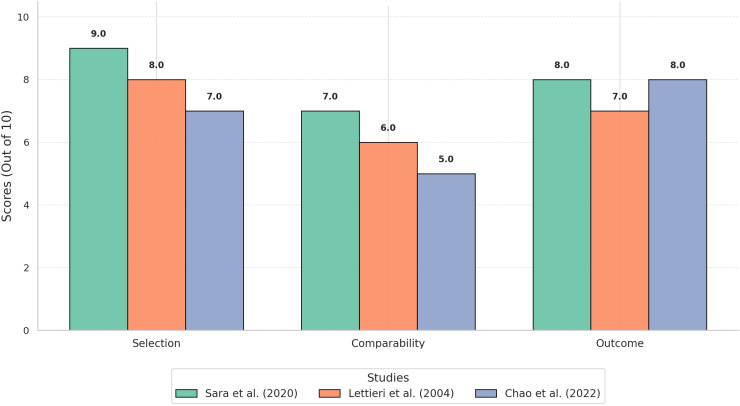
Assessment of study quality using the Newcastle-Ottawa Scale (NOS). This figure presents the quality assessment of the included studies using the Newcastle-Ottawa Scale (NOS). The studies are categorized based on their scores in three domains: selection, comparability, and outcome, with studies receiving scores of ≥7 considered high-quality, 4–6 as medium-quality, and <4 as low-quality.

The quality of the included studies was assessed using the Newcastle-Ottawa Scale (NOS). Scores ranged from 7 to 9, indicating medium to high methodological quality. All studies demonstrated strong sample representativeness and reliable diagnostic methods. However, some studies received lower scores in the comparability domain because they did not account for potential confounding factors. The detailed NOS assessment is shown in Fig 2.

### 3.3. Prevalence of leptospirosis among soldiers

The reported prevalence of leptospirosis among military personnel varied widely across the included studies. Sara et al. (2020) in Northeastern Malaysia reported the highest prevalence (16.2%), whereas Lettieri et al. (2004) in Hawaii reported a lower prevalence of 1.4%. Chao et al. (2022) in Honduras reported a prevalence of 11.6%. These variations may reflect differences in geographic regions, environmental exposures, and diagnostic methods.

Studies conducted in tropical regions, such as Malaysia and Honduras, reported higher prevalence rates, likely due to environmental factors conducive to Leptospira transmission. Additionally, the use of more sensitive diagnostic techniques, such as combined ELISA and MAT, may have contributed to higher reported rates.

## 4. Discussion

Leptospirosis is one of the main zoonotic diseases worldwide due to its high morbidity and mortality, with approximately one million cases and 59,000 deaths each year in humans and a significant impact on animals, particularly in tropical and subtropical zones [14,15]. This systematic review is the first to determine the prevalence of leptospirosis among military personnel. The seroprevalence of leptospirosis varies among professions and is influenced by several factors, such as region, weather, and climate [16]. The results of the study can also be influenced by the characteristics of the selected participants, such as gender.

The significance of leptospirosis as a public health concern among military personnel is underscored by numerous outbreak reports. For instance, a study by Hadad et al. (2006) in Israel documented an outbreak involving 7 members of a team of 27 troops following a military exercise near the Jordan River [17]. The causative organism was identified as *Leptospira interrogans* serovar Hardjo. Similarly, Lupi et al. (2020) reported an outbreak in Brazil involving military personnel, with two cases requiring hospitalization after field training exercises [18]. The affected individuals presented with symptoms such as acute meningoencephalitis, respiratory illness, and skin rash. The implicated serovars were *Icterohaemorrhagiae*, *Hebdomadis*, *Patoc*, and *Cynopteri*.

Other notable outbreaks include one described by Katz et al. (1997) in Oahu, Hawaii. In December 1992, a common-source waterborne outbreak led to two confirmed cases and 18 additional exposed individuals [19]. Burns et al. (2016) reported a case in Birmingham, UK, involving a 32-year-old soldier who returned from Borneo with fever and acute kidney injury caused by leptospirosis [20].

Dierks et al. (2018) documented an outbreak among Marine trainees in Okinawa, Japan, with 81 cases identified among 239 personnel, resulting in an attack rate of 33.9% [21]. Neela et al. (2019) reported cases among reserve military recruits in Hulu Perdik, Malaysia. Of the 12 recruits presenting with febrile illness, 2 (16.6%) were confirmed positive for leptospirosis using the MAT [22]. Gentile et al. (2020) reported 88 cases of leptospirosis among members of the French Armed Forces. Of these, 15 cases occurred in continental France, while 73 cases were reported overseas, including 42 cases in the French West Indies [23]. Russell et al. (2003) investigated an outbreak of acute febrile illness among Peruvian military recruits, reporting 78 cases out of 193 recruits. Of these, 72 were confirmed to have acute leptospirosis using the MAT [24].

Chen et al. (2019) conducted a study involving US Marines stationed in Japan. Out of 1,000 post-tour samples tested, 85 were positive for Leptospira-specific immunoglobulin G, indicating past exposure to leptospirosis [25]. The prevalence of leptospirosis antibodies in Pakistani military personnel was found to be between 1% and 6% [26].

We did not include in our systematic review article investigating military personnel working with animals. Among 65 active military personnel, 54 (83.1%) tested positive for leptospirosis infection because this group is likely to have a higher rate of infection due to contact with animals [27].

Leptospirosis is diagnosed by detecting bacteria in blood and urine samples using direct dark-field microscopy, culturing *Leptospira* with culture methods, detecting the *Leptospira* genome through molecular methods (PCR), or identifying antibodies using serological tests such as the MAT, ELISA, rapid diagnostic tests, dot-ELISA, and immunochromatographic assays [28,29].

The MAT is considered the gold standard serological test for detecting human leptospirosis [28]. In studies analyzing prevalence, two major types of serological reactions were used: ELISA and MAT. ELISA tests can be designed to detect specific antigens, such as LipL32, which has shown high sensitivity and specificity for IgG antibodies [30,31]. Interestingly, pooled meta-analysis indicates that, based on ELISA, IgM antibodies are more specific than IgG antibodies for leptospirosis [32]. This may be because other febrile illnesses can cause a non-specific rise in IgG, leading to false positives. IgG-ELISA has also shown better performance than MAT in monitoring IgG responses to *Leptospira* vaccines in cattle [33]. Among the three included studies: Sara et al. [34] used ELISA for serological screening, confirmed by MAT; Lettieri et al. [35] used MAT for 16 serovars; and Chao et al. [36] used ELISA.

Leptospirosis in soldiers is not well studied, even though the military is an important part of every country. Soldiers often work in areas where the risk of infection is high, especially in tropical and subtropical regions [37]. Compared to other infectious diseases, such as malaria and dengue fever, leptospirosis has received less attention. This may be because its symptoms are similar to those of other illnesses, such as scrub typhus, dengue, and malaria, making it difficult to diagnose [25].

Laboratory diagnosis of leptospirosis requires special media for pathogen isolation and specific methods for serological testing [38]. The limited availability of laboratory support for diagnosing leptospirosis likely contributes to underreporting. Additionally, in endemic regions, many infections may cause only mild symptoms, meaning that laboratory tests are often not performed [39].

For diagnosing leptospirosis in fieldwork during war, ELISA is commonly used instead of the standard MAT because it is easier to perform and allows for testing many samples at once [40,41]. The ELISA assay uses four highly purified recombinant antigens that are found only in pathogenic Leptospira species and are highly conserved across different pathogenic serovars.

For better diagnosis, an IgM dipstick assay could be used instead of routine ELISA or MAT [42]. The IgM dipstick assay has shown higher sensitivity than other tests, such as IgM ELISA and indirect hemagglutination assay (IHA). Studies have reported an overall sensitivity ranging from 1.8% to 75% and a specificity ranging from 52.3% to 97.7% when evaluating five different rapid tests [43]. These results vary widely. Margarita Arboleda et al. recommend using the PCR method due to its high sensitivity. They also suggest that, due to the COVID-19 pandemic, PCR testing has become more widely available [44].

Another important issue is the lack of awareness about leptospirosis, both among military personnel and the general public. Despite the risk of severe complications and even death, many soldiers may not be aware of how the disease spreads or how to prevent it. This is different from malaria, for which military personnel often receive detailed training and preventive medication. Adding leptospirosis awareness programs to military health education and promoting protective measures, such as wearing waterproof gear and maintaining good hygiene, could help lower infection rates.

Specific prevention strategies include vaccination and chemoprophylaxis. Research on human vaccines for leptospirosis is progressing, and some countries, such as Japan, Cuba, France, and China, have tested and approved vaccines for use in their populations [45]. Regarding antibiotic prevention, some studies suggest that taking doxycycline weekly before and during exposure can help prevent leptospirosis in endemic areas. However, a recent study on a leptospirosis outbreak among US Marines in Okinawa found no significant difference in infection rates between those who took doxycycline before or after exposure and those who did not [21]. The study concluded that a higher risk of infection was connected to internal exposure, such as drinking contaminated water or having open wounds that came into contact with infected water.

Health organizations play a pivotal role in addressing these gaps. International bodies, such as the World Health Organization (WHO) and the Centers for Disease Control and Prevention (CDC), can provide technical guidance, facilitate global surveillance, and support research initiatives. Military medical teams and defense health agencies should work together with public health groups to create better reporting systems and improve testing in high-risk areas. These actions can help lower the number of leptospirosis cases in soldiers, improving their health and making military operations more effective.

This systematic review has several limitations that need to be considered. First, there is a possibility of publication bias, as studies with negative or inconclusive results may not have been published or included. Second, variations in diagnostic methods across studies could influence the comparability of results, as different serological or molecular techniques may lead to varying prevalence estimates. Additionally, the included studies are primarily from specific geographic regions (such as tropical and subtropical areas), which may limit the generalizability of the findings to other regions or populations. Lastly, the retrospective nature of some studies and incomplete reporting of data, including participant demographics, might affect the accuracy and reliability of the conclusions drawn from the results.

## Supporting information

S1 File PRISMA checklist. (DOCX)

S2 File Search strategy. (DOCX)
