# Supplementary material for: Prevalence of leptospirosis among soldiers: A systematic review
Source: PLoS Negl Trop Dis. 2025 Mar 17;19(3):e0012927. doi: 10.1371/journal.pntd.0012927 (PMC12165700; doi:10.1371/journal.pntd.0012927)
Supplement: S2 File — (DOCX) [file pntd.0012927.s002.docx]

**S1 Search Strategy**

**Web of Science**

TS=(leptospirosis OR Leptospira) AND TS=(soldier* OR military OR "armed forces" OR "defense personnel" OR troop* OR army OR navy OR "air force" OR "defense services") AND TS=(prevalence OR seroprevalence OR epidemiology OR incidence)

**PubMed**

(("Leptospirosis"[MeSH Terms] OR "Leptospira"[Title/Abstract]) AND (soldier*[Title/Abstract] OR military[MeSH Terms] OR "armed forces"[Title/Abstract] OR "defense personnel"[Title/Abstract] OR troop*[Title/Abstract] OR army[Title/Abstract] OR navy[Title/Abstract] OR "air force"[Title/Abstract] OR "defense services"[Title/Abstract]) AND (prevalence[MeSH Terms] OR "seroprevalence"[Title/Abstract] OR epidemiology[MeSH Terms] OR incidence[Title/Abstract]))

**Scopus**

TITLE-ABS-KEY ( leptospirosis OR leptospira ) AND TITLE-ABS-KEY ( soldier* OR military OR "armed forces" OR "defense personnel" OR troop* OR army OR navy OR "air force" OR "defense services" ) AND TITLE-ABS-KEY ( prevalence OR seroprevalence OR epidemiology OR incidence ) AND PUBYEAR > 1999 AND PUBYEAR < 2025 AND ( LIMIT-TO ( DOCTYPE , "ar" ) )
